# Comparison of Point-of-Care Ultrasound Guidance and the Traditional Approach in Performing Simulated Emergent Cricothyroidotomy Among Emergency Medicine Physicians, Residents, and Students

**DOI:** 10.7759/cureus.71015

**Published:** 2024-10-07

**Authors:** Marc Dotson, Stephanie Thompson, Gurmaninder P Singh, Scott Area

**Affiliations:** 1 Emergency Medicine, Charleston Area Medical Center, Charleston, USA; 2 Research, Charleston Area Medical Center, Charleston, USA; 3 Graduate Medical Education, Charleston Area Medical Center, Charleston, USA

**Keywords:** cricothyroidotomy, difficult airway management, emergency medicine, simulation, ultrasound

## Abstract

Objective

Emergency cricothyroidotomy (EC) is a rare procedure used to establish airway access when both endotracheal intubation and bag-mask ventilation have failed. Point-of-care ultrasound (POCUS) has been proposed as an adjunct to aid in identifying anatomical landmarks. However, its impact on emergency physicians when performing EC remains unclear. This study compared emergency physician and student confidence, preference, and procedure time between POCUS and traditional palpation for EC.

Methods

Our study utilized a randomized controlled crossover design. Emergency medicine providers attended a didactic lecture demonstrating traditional palpation and POCUS techniques for EC. The participants were randomly allocated to two simulation groups. One group started with the palpation of landmarks, while the other group started with POCUS landmark identification. A randomized crossover design was employed, and in a subsequent simulation, the reverse technique was utilized. Time to anatomic landmark identification was recorded. Participants completed a survey assessing their confidence and preference. Procedure times and confidence level differences were examined via Wilcoxon signed-rank tests.

Results

Sixteen participants completed the survey and were included in the final analysis. All participants successfully performed EC with the two landmark identification techniques. There was no significant difference in self-reported confidence between POCUS and palpation (p = 0.17). Landmark identification by palpation was significantly faster than POCUS (p = 0.001). A total of 10 (63%) participants preferred palpation over POCUS.

Conclusions

Both POCUS and traditional palpation were effective in identifying anatomical landmarks for EC. Although palpation was significantly quicker, confidence and preference between the two techniques were similar. The results suggest that both approaches can benefit clinical practice, depending on the context and provider familiarity. Further studies with larger cohorts and real-world scenarios are recommended to explore the effectiveness and safety of POCUS in EC.

## Introduction

Background 

Emergency cricothyroidotomy (EC) is a procedure to establish airway access in patients for whom the medical provider cannot ventilate via other means. The scenario of “can't intubate, can't oxygenate” (CICO) describes an emergency in which endotracheal intubation and bag-mask ventilation have both failed. A surgical airway must be created to prevent further oxygen desaturation and subsequent patient morbidity or mortality [1]. Though cricothyroidotomy techniques vary, airway access in this procedure centers around creating a passage through the cricothyroid membrane and anterior trachea, allowing for the passage of an airway device [2]. 

EC is a critical yet rare procedure. A 2018 study assessing advanced airway management in 401 emergency medicine service agencies found that out of 57,209 patients requiring advanced airway management procedures, only 0.5% underwent EC [3]. Due to the procedure’s rarity, medical providers are typically trained how to perform EC by direct visual identification of anatomical landmarks on high-fidelity mannequin models, porcine tracheas, or cadaver models and may have a comparatively small amount of experience performing the procedure on patients [4]. Additionally, misidentifying the cricothyroid membrane through digital palpation is a frequent mistake, causing unsuccessful cricothyrotomies and negative consequences [5].

Ultrasonography, commonly known as ultrasound imaging, refers to using high-frequency sound waves to view anatomical structures in the body [6]. Ultrasound imaging allows clinicians to identify anatomical structures more rapidly than alternative imaging modalities in real time. When performed at the bedside, this is known as point-of-care ultrasound (POCUS) and has been shown to provide positive results during procedures, such as obtaining bedside peripheral intravenous access, by allowing for the visualization of veins that could not otherwise be identified [7]. The use of POCUS has demonstrated improved identification of relevant anatomy, insertion of airway device success rates, and lower complication rates in cadaveric models [8,9]. In patients experiencing cardiac arrest, POCUS is often used to identify the presence or absence of cardiac activity during pulse checks and is superior to pulse detection by palpation [10]. 

Importance 

Despite its benefits, there are potential disadvantages of utilizing ultrasound in rapidly deteriorating patients with life-threatening conditions. In a study of 110 cardiac arrest patients receiving cardiopulmonary resuscitation (CPR), Clattenburg et al. found that the use of POCUS was associated with longer delays in restarting CPR after pulse checks [11]. While this study did not endeavor to study the use of POCUS in patients with cardiac arrest, the delay in treatment incurred by the use of POCUS is often anecdotally cited as a reason some providers choose not to use this method. 

POCUS can assist providers in identifying the cricothyroid membrane when performing EC, especially in scenarios where direct visual identification or identification through palpation may be difficult [8,9,12,13]. Nicholls et al. found that in a study of emergency physicians, 50 (100%) could identify the cricothyroid membrane on ultrasound in live patients after completing a standardized ultrasound training course [8]. Additionally, Siddiqui et al. found that in cadavers with poorly defined neck anatomy, providers using ultrasound guidance in the EC procedure were 5.6 times more likely to correctly insert an airway device when landmarks could not be palpated compared to those not using ultrasound [9]. 

Goals of this investigation 

A lack of technique comparison within the same providers often limits studies exploring the usage of POCUS in EC. Most studies on ultrasound-guided cricothyroidotomy have explored accuracy, anatomical landmarks, and techniques [8,12,13]. Also, most studies comparing techniques have been in anesthesia literature [9,14,15]. Therefore, this study examined emergency physician performance and preference when trained in POCUS and traditional palpation methods for anatomical landmark identification in EC. After completing didactic and simulation training, our goal was to allow the participating attendings, residents, and students to perform the procedure utilizing both approaches and compare their success rate, procedure times, comfort level, and technique preference. With the hypothesis that using POCUS will increase the success rate, this study contributes to the growing evidence that POCUS is advantageous in performing EC.

## Materials and methods

Study design, setting, and participants**

**Figure 1:**
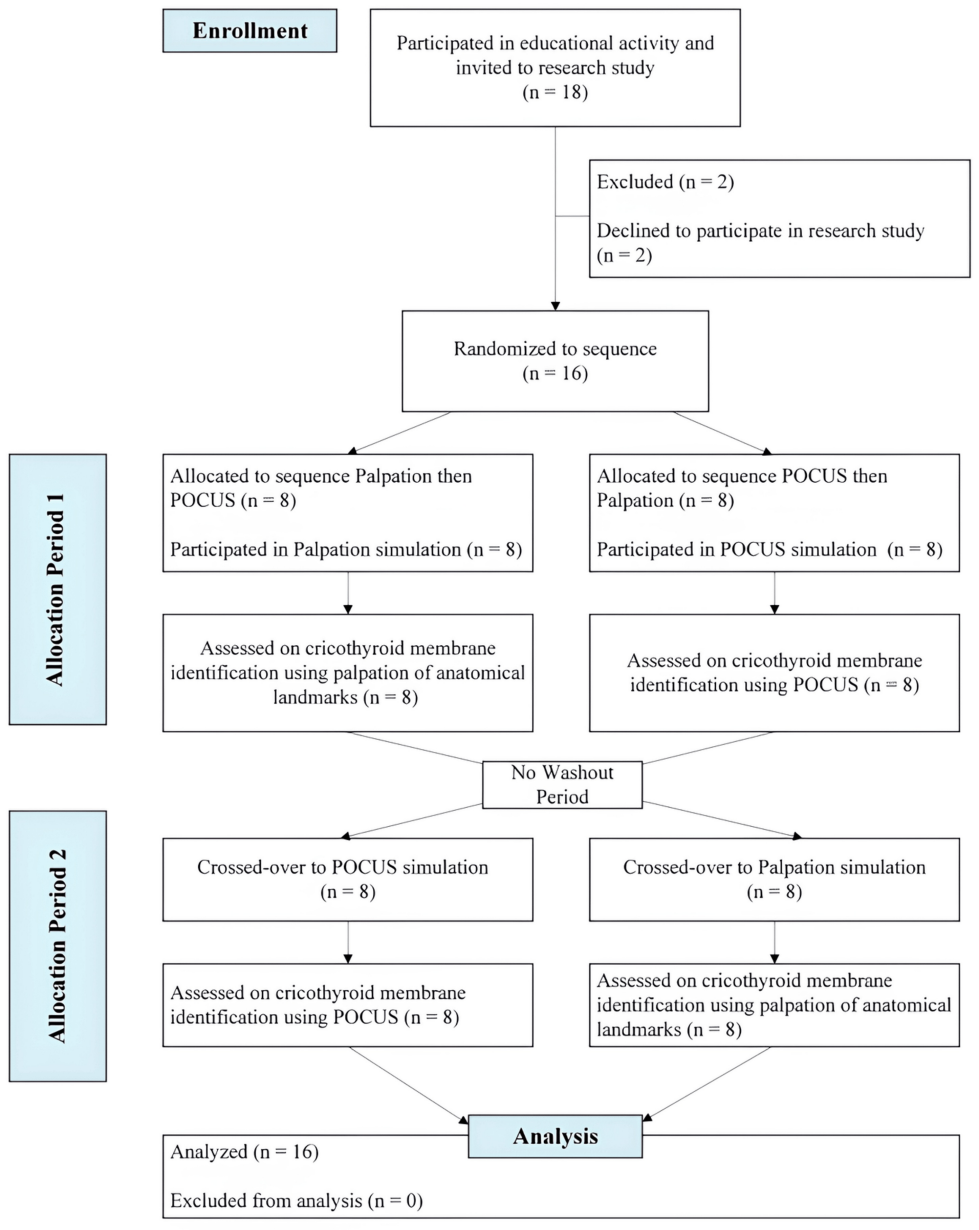
CONSORT flow diagram showing the number of participants through each stage of the randomized crossover study CONSORT: Consolidated Standards of Reporting Trials; POCUS: Point-of-care ultrasound

This study used a randomized controlled crossover design (Figure 1). Emergency medicine attendings and resident physicians at our health care system and medical students rotating in the Emergency Medicine department were invited to a 30-minute PowerPoint-based didactic lecture teaching the EC procedure and anatomical landmark identification via palpation and POCUS. Participants were told the learning and research objectives, and participation was stated to be voluntary. Verbal consent was obtained before starting the training activity. The Charleston Area Medical Center/West Virginia University Charleston Division Institutional Review Board approved all study aspects (approval number 23-930). 

Anatomical landmark identification was taught, demonstrated, and practiced on high-fidelity mannequin models and volunteer, living human models. After the training course ended, participants were randomly separated into two simulation groups: 1.) those whose first simulation utilized identification by palpation of anatomical landmarks and 2.) those whose first simulation utilized POCUS. Participants were then given a single attempt to demonstrate the use of palpation or POCUS-guided cricothyroidotomy procedures separately on mannequin models, with each simulation having a time limit of 120 seconds. 

Measures**

To control for technique bias, each group performed the opposite technique on a second cricothyroidotomy simulation. The instructor recorded the participants’ performance during the simulation on a grading sheet. Variables included procedure success (achieving tracheal cannulation within 120 seconds), time to cricothyroid membrane identification (in seconds), and time to tracheal cannulation (in seconds). 

Procedure 

After completing both simulations, participants completed an electronic REDCap questionnaire assessing their comfort and familiarity with visual and ultrasound-guided EC. Self-reported confidence was assessed by using a 5-point Likert scale with 1 = Strongly disagree and 5 = Strongly agree for the statements “After didactic training and simulation, I feel confident I can identify anatomical landmarks on a live patient using ultrasound“ and “After didactic training and simulation, I feel confident I can identify anatomical landmarks visually on a live patient.” Additionally, participants recorded which method was their preferred technique by answering the question “If you needed to perform the Emergency Cricothyroidotomy procedure on a live patient, which landmark identification technique would you prefer to use?” 

Analysis 

IBM SPSS Statistics for Windows, Version 19 (Released 2010; IBM Corp., Armonk, New York, United States) was used to analyze the data. Basic descriptive statistics included medians and interquartile ranges (IQR) for ordinal Likert scale values and procedure times as they were not normally distributed, and proportions and frequencies for categorical variables. We compared procedure times and provider-reported confidence within subjects for traditional palpation and POCUS in EC procedures using the Wilcoxon signed-rank test. Fisher exact test analysis compared technique preference rates between learners (students and residents) and attendings. To compare possible crossover effects, we evaluated the differences in confidence and procedure times according to the assigned technique sequence (palpation then POCUS vs. POCUS then palpation) using Mann-Whiteney U analyses. A p-value of < 0.05 was used to determine statistical significance.

## Results

Eighteen participants attended the PowerPoint-based EC didactic lecture and the associated simulations. Sixteen participants completed the post-simulation survey and were included in the final analysis. The participants included three third-year medical students (19%), six resident physicians (37.5%), six attending physicians (37.5%), and one (6%) current position not recorded. A total of 14 (88%) participants had prior experience or training in palpation, 4 (25%) had prior training with POCUS, and 2 (13%) had no previous training in either method. Eight participants (50%) performed palpation cricothyroidotomy followed by POCUS, while the other 8 (50%) began with POCUS followed by traditional palpation. 

All 16 (100%) participants successfully performed EC utilizing each palpation and POCUS anatomical landmark identification method. The primary outcome compared self-reported confidence in identifying the cricothyroid membrane during the EC procedure. The results indicated no significant difference in confidence between identification by palpation (4.0, 3.0-4.8) vs. POCUS (3.5, 3.0-4.0, p = 0.16; see Table 1). 

**Table 1:** Comparison of point-of-care ultrasound guidance and the traditional, palpation approach in emergent cricothyroidotomy. The data has been represented as medians (range), number (%), and p<0.05. POCUS: Point-of-care ultrasound

	Palpation	POCUS t	p-value
Successfully performed emergent cricothyroidotomy	16 (100%)	16 (100%)	
Confidence in identifying anatomical landmarks needed for emergent cricothyroidotomy (1=Strongly disagree and 5=Strongly agree)	4.0 (3.0-4.3)	3.5 (3.0-4.0)	0.16
Time required to identify cricothyroid membrane, seconds	3.1 (2.5-5.2)	15.0 (10.4-29.2)	0.001
Time required to perform emergent cricothyroidotomy successfully, seconds	29.3 (22.9-37.5)	42.7 (34.9-75.6)	0.004

Secondary outcomes included procedure times. Cricothyroid membrane identification by palpation was significantly faster compared to POCUS (3.1, 2.5-5.6 vs. 15.0, 10.2-36.4 seconds, p = 0.001), as was tracheal cannulation completion (29.3, 22.7-38.5 vs. 42.7, 34.9-75.6 seconds, p = 0.003), indicating that POCUS required more time. 

Additionally, we assessed provider preference for POCUS versus palpation for cricothyroid membrane identification. Overall, 10 (63%) participants preferred identification by palpation over POCUS. The level of experience did not associate technique preference with 6 of 9 (67%) learners preferred palpation vs. 3 of 6 (50%) attendings preferred palpation for landmark identification (p = 0.62). Thus, learners and experienced providers exhibited similar preferences for the two techniques. 

We examined whether provider confidence, procedure times, and preference were influenced by which landmark identification technique was completed first (Table 2). In an analysis of potential carryover effects, there were no significant differences in these outcomes of interest between clinicians who started with palpation versus those who started with POCUS.

**Table 2:** Confidence, procedure times, and preference according to the assigned technique sequence. The data has been represented as medians (range), number (%), and p<0.05. POCUS: Point-of-care ultrasound

	Palpation then POCUS cohort n =8	POCUS then Palpation cohort n=8	p-value
“After didactic training and simulation, I feel confident I can identify anatomical landmarks visually on a live patient” (1=Strongly disagree and 5=Strongly agree)	4.0 (2.3-4.8)	4.0 (3.3-4.8)	0.65
“After didactic training and simulation, I feel confident I can identify anatomical landmarks on a live patient using ultrasound“ (1=Strongly disagree and 5=Strongly agree)	3.0 (2.0-4.0)	4.0 (3.0-4.8)	0.19
Time required to identify cricothyroid membrane using palpation, seconds	3.1 (2.3-7.5)	3.5 (2.5-4.9)	0.65
Time required to identify cricothyroid membrane using POCUS, seconds	15.0 (8.5-25.3)	17.2 (10.2-53.7)	0.88
Time required to successfully perform emergent cricothyroidotomy using palpation, seconds	30.5 (23.3-46.8)	29.3 (19.3-36.7)	0.44
Time required to successfully perform emergent cricothyroidotomy using POCUS, seconds	41.9 (33.0-65.3)	54.2 (34.0-90.7)	0.57
If you needed to perform the Emergency Cricothyroidotomy procedure on a live patient, which landmark identification technique would you prefer to use?			0.61
Palpation	4 (50%)	6 (75%)	
Pocus	4 (50%)	2 (25%)	

## Discussion

This study compared the effectiveness of POCUS and traditional palpation in identifying anatomical landmarks for EC among emergency attendings, residents, and medical students. Our findings provide insights into POCUS's potential benefits and limitations in emergency airway management.

The primary outcome of our study was self-reported confidence in identifying the cricothyroid membrane during EC. Our results demonstrated no significant difference in confidence levels between participants using palpation and those using POCUS. This finding suggests that with proper training, emergency medicine attendings and trainees can achieve similar confidence regardless of the technique employed. Our findings add to the evidence of the value of incorporating POCUS into emergency airway management, particularly during preparation for EC [16]. On the other hand, we found that 10 (63%) participants preferred palpation over POCUS. This preference may come from the perception that palpation is quicker and less dependent upon additional equipment and training. Existing literature, however, supports that POCUS increases accuracy over palpation in identifying the cricothyroid membrane [8,9,15,17].

The concern about delays in patient care when using POCUS is valid, especially in specific emergencies like cardiac arrest, where ultrasound has been shown to cause delays in critical interventions [11,18]. One of the secondary outcomes of our study was the time required to identify the cricothyroid membrane and tracheal cannulation completion. Our findings suggest that palpation is faster at identifying CTM, and when EC is performed with POCUS, it requires more time. This could be due to various factors but is likely related to equipment setup and interpretation of the image. Therefore, the authors believe that a clinician using POCUS to identify the membrane should use it early in the patient's preprocedural airway assessment and before a CICO situation, specifically in patients with abnormal neck anatomy or obesity [9,14,15].

Our study examined whether experience level influenced the choice between palpation or POCUS for identifying anatomical landmarks. We found no significant difference in technique preference based on provider experience level, suggesting that preference for either method is independent of experience.

Finally, provider confidence and procedure times did not vary based on which technique was performed first during the assessment. This indicates that the order in which techniques are introduced during training does not affect provider performance, suggesting flexibility for educators in designing training programs.

Limitations

Some limitations were present in our study. First, the small sample size limits the findings' generalizability and reduces power. However, the significance of the p-values suggests that the observed effect is statistically meaningful within the study's context. Secondly, the simulation-based nature of the study, while useful for training, cannot perfectly replicate real-life emergency scenarios, where factors such as stress, patient anatomy, and environmental constraints can impact outcomes. While our study focused on measuring confidence, preference, and procedure time, due to taking place in a simulation environment, it could not address other critical factors such as the accuracy of the procedure, patient outcomes, or the rate of complications. Future studies involving larger cohorts and real-world scenarios could provide a more comprehensive understanding of the effectiveness and practicality of POCUS use in EC.

## Conclusions

In conclusion, our findings suggest that both traditional palpation and POCUS can effectively identify anatomical landmarks for EC. While palpation was a faster technique than POCUS, there were no significant differences in confidence among participants, suggesting that both methods can be valuable in clinical practice. Technique choice should depend on the specific circumstances and provider familiarity. Further research is needed to explore the real-world application and outcomes of these techniques in emergency airway management. 
